# Awareness of occupational hazards and associated factors among welders in Lideta Sub-City, Addis Ababa, Ethiopia

**DOI:** 10.1186/s12995-016-0105-x

**Published:** 2016-04-05

**Authors:** Sebsibe Tadesse, Kassahun Bezabih, Bikes Destaw, Yalemzewod Assefa

**Affiliations:** Institute of Public Health, the University of Gondar, Gondar, Ethiopia; City Government of Addis Ababa Health Bureau, Addis Ababa, Ethiopia

**Keywords:** Hazard awareness, Welding, Workplace factors, Workplace safety

## Abstract

**Background:**

Welding is a manufacturing industry where workers could be exposed to several hazards. However, there is a dearth of studies clarifying the situation in Ethiopia. The present study determined the level of awareness of occupational hazards and associated factors among welding employees at Lideta Sub-City, Addis Ababa, Ethiopia.

**Methods:**

A work site-based cross-sectional study was conducted among welding employees Lideta Sub-City, Addis Ababa, Ethiopia from April to May 2015. Stratified sampling followed by simple random sampling techniques was used to select the study participants. A pilot tested and structured questionnaire was used to collect data. Multivariable analyses were employed to see the effect of explanatory variables on workers’ awareness of occupational hazards.

**Results:**

According to our criteria of awareness 86.5 % of surveyed workers were aware of occupational hazards. A higher work experience, presence of work regulation, job satisfaction, being married, being single, and a higher educational status were factors significantly associated with workers’ awareness of occupational hazards.

**Conclusion:**

This study revealed that the level of awareness of occupational hazards among welders was high. However, this does not mean that there will be no need for further strengthening of the safety measures as significant proportions of the workers still had low awareness. Interventions to boost workers awareness of occupational hazards should focus on areas, such as provision of safety trainings, promotion of safety advocacy, and enforcement of appropriate workplace safety regulation.

## Background

The number of occupational accidents and diseases are increasing in developing countries. It has been estimated that over 120 million occupational accidents with over 200,000 fatalities occur each year in these countries [1]. Subsaharan Africa appears to have the greatest rate followed by Asia [2]. There were an estimated 42 million occupational accidents with over 54,000 fatalities annually [3]. In Ethiopia there were an estimated 4.3 million occupational accidents with over 5596 fatalities annually. This gave accident and fatality rate of 16,426 and 21.5 per 100,000 workers, respectively [3].

Welding is one of the occupations that contribute to work-related accidents and diseases in the context to developing countries [4]. The process remains the most common method of joining metals today and is a part of the art of metal fabrication that involves the building of metal structures by cutting, bending and joining. Polishing, painting or coating of the metal pieces also goes along with the other processes [3, 4]. Welding hazards such as the bright and blinding light of the welding arc, the hazardous composition of the welding fumes, the sharp metal edges as well as the hot and flying molten metal particles, fast moving machinery, noise, and vibration may lead to acute and chronic health effects [5, 6]. The acute symptoms may consist of metal fume fever (flu-like symptoms with alternating chills and high fever that last for a few days), irritation of the eyes, nose, chest and respiratory tract causing cough, wheezing, breathlessness, bronchitis, pulmonary edema, pneumonitis and gastrointestinal effects, such as nausea, loss of appetite, vomiting, cramps and slow digestion [7, 8]. Chronic health effects include increased risk of lung cancer, cancer of the larynx and urinary tract, hypertension, varieties of respiratory problems, such as bronchitis, asthma, pneumonia, emphysema, pneumoconiosis, decreased lung capacity, silicosis and siderosis. Other chronic effects include heart and skin diseases, hearing loss, chronic gastritis, gastro-duodenitis, ulcer of the stomach and small-intestine, kidney damage, and damage to the reproductive system leading to reduction in sperm count and fecundity. Musculoskeletal problems, such as back injuries, shoulder pain, tendonitis, reduced muscle strength, carpal tunnel syndrome, white finger, and knee joint diseases are the other health problems [9–12]. Physical and accidental risks, like burns, cuts, lacerations, and fall injuries are also common [4, 13].

Industrial safety and health problems are becoming major challenges in Ethiopia because of low occupational hazards awareness, lack of workplace safety and health policy, and inefficient safety management system. Due to these employers, workers and the government are losing measurable costs. There is an information gap on occupational hazards in welding industries in the country. Therefore, the aim of this research is to assess the level of awareness of occupational hazards and associated factors among welders at Lideta Sub-City, Addis Ababa, Ethiopia. Such information is vital in understanding the extent of the problem and may be useful when designing intervention strategies targeted at promoting and upholding good health and safety standards in this important working group.

## Methods

### Study design, area and period

A work site-based cross-sectional study was conducted to assess level of awareness and factors associated with occupational hazards among welders at Lideta Sub-City, Addis Abba, the capital city of Ethiopia, from April to May 2015.

### Participants and data collection

All employees who directly involved in the process of welding were included in the study until the required sample size was obtained. Workers who were absent from work due to different reasons during the time of data collection were excluded from the study. A pilot tested and structured interview questionnaire was used to collect the data. Six trained people with first degree in public health administered the questionnaire. The questionnaire contained detailed information on socio-demographic, behavioral and workplace factors that could have association with hazard awareness.

### Sample size calculation

A single population proportion to size formula was used to determine the sample size of the study. The total sample size was determined to be 567 by taking 95 % confidence interval, 34.2 % expected proportion (P) [14], 4 % margin of error (W), 5 % non-response rate. That is, Sample size $=\kern0.5em \frac{{\left(\frac{z\alpha }{2}\right)}^2*P\left(1-P\right)}{W^2} = \frac{3.842*0.225}{0.0016} \approx 540$. Adding 5 % non-response rate gives 567.

### Sampling procedure

Stratified sampling followed by simple random sampling techniques was used to select the study participants. That is, the industries were stratified into three scales, namely large (employed ≥50 workers), medium (employed 10–49 workers) and small (<10 workers) [15]. Then, the total of 567 samples was proportionally allocated to each industry. That is, 99 to large scale (N1 = 184), 195 to medium scale (N2 = 363), and 273 to small scale (N3 = 507). The participants were drawn from the industries’ list of workers using simple random sampling.

### Data quality control

The training of data collectors and supervisors emphasized issues such as data collection instrument, field methods, inclusion–exclusion criteria, and record keeping. The investigators and supervisors coordinated the interview process, spot-checked and reviewed the completed questionnaire on a daily basis to ensure the completeness and consistency of the data collected. The interview questionnaire was pilot tested on 29 respondents in order to identify potential problem areas, unanticipated interpretations, and cultural objections to any of the questions.

### Data management and statistical analyses

Data entered and cleaned using Epi info version 3.5.1 statistical software were analyzed on SPSS version 20. Frequency distribution, mean, standard deviation, and percentage, were employed for most variables. All independent variables were fitted separately into bivariate logistic model to evaluate the degree of association with hazard awareness. Then, variables with a *p*-value < 0.20 were exported to multivariable logistic regression model to control confounders. The odds ratio (OR) with a 95 % confidence interval (CI) was used to test the statistical significance of variables.

### Operational definitions

#### Awareness of occupational hazards

Summary score was calculated for the participants’ awareness of hazards that were potentially related to their work based on ten questions. These were: can weld cause 1) arc eye injury; 2) foreign body enter into eye; 3) breathlessness 4) chronic cough; 5) metallic fume fever; 6) injuries to the body; 7) burns to the body; 8) explosion; 9) back pain; 10) hearing impairment. The mean score for awareness of hazards was taken as a cut-off point and those who scored above the mean were considered as having awareness.

#### Job satisfaction

Was a self-reported felling of participants about their job as it was pleasurable for them.

#### Personal protective equipment (PPE)

Workers were observed for their utilization of specialized clothing or equipment for protection against health and safety hazards at the time of interview. The observation was made for about 5 min just before starting administration of the questionnaire.

#### Permanent employee

Any contract of employment between employee and employer concluded for an indefinite period [16].

#### Temporary employee

Any employment contract between employee and employer made for definite period [16].

#### Ethical considerations

The study protocol was reviewed and approved by the Institutional Review Board of the University of Gondar via the Institute of Public Health. Permission was obtained from Lideta Sub-City’s large, medium and small scale industry offices prior to data collection. Study participants were interviewed after informed written consent was obtained. They were also informed that their participation was voluntary and that they could withdraw from the interview at any time without consequences. The participants were assured that their responses would be treated confidentially through the use of strict coding measures.

## Results

### Socio-demographic characteristics

A total of 555 employees completed the questionnaire making response rate 97.9 %. Of whom 98.2 % were males. The majority, 85.9 %, of the employees belonged to the age group of 30–53 years. Half, 48.8 %, of them were married. Regarding educational status 44.0 % attended secondary education (Table 1).

**Table 1:** Socio-demographic characteristics of welders at Lideta Sub-City, Addis Ababa, Ethiopia, 2015

Variables	Number	Percent
Sex		
Male	545	98.2
Female	10	1.8
Age (in years)		
18–29	53	9.5
30–41	332	59.8
42–53	145	26.1
≥54	25	4.5
Marital Status		
Single	263	47.4
Married	271	48.8
Divorced	21	3.8
Educational status		
Primary	83	15.0
Secondary	244	44.0
Certificate and above	228	41.1

### Workplace and behavioral characteristics

Three-fourths, 75.1 %, of the participants were permanent employees. About thirty eight percent served for less than 5 to 9 years. Regarding hours spent on work 95.9 % of the employees had worked for more than 40 h per week. Nearly three-fourths, 72.1 %, were satisfied with their job. Sixty percent did not attend any kind of safety training. The majority, 83.2 and 82.0 %, complained lack of work shift and safety supervision during work, respectively. About 93.2 % used at least one kind of PPE during work. The majority, 91.8, 85.4 and 61.3 %, of them used goggle, coverall, and safety shoe, respectively. Fifty eight percent drank alcohol followed by 44.0 % smoked cigarette and 39.5 % chewed khat (Table 2).

**Table 2:** Workplace and behavioral characteristics of welders at Lideta Sub-City, Addis Ababa, Ethiopia, 2015

Variables	Number	Percent
Employment pattern		
Permanent	417	75.1
Temporary	108	19.5
Work experience (in years)		
≤5	151	27.2
5–9	208	37.5
10–14	99	17.8
≥15	97	17.5
Hours worked per week		
≤40	23	4.1
>40	532	95.9
Job satisfaction		
Satisfied	400	72.1
Dissatisfied	155	27.9
Attended safety training		
Yes	220	39.6
No	335	60.4
Safety supervision		
Yes	99	17.0
No	456	82.0
Work shift		
Yes	92	16.6
No	463	83.2
Used PPE		
Yes	194	35.0
No	361	65.0
Type of PPE used		
Goggle	509	91.8
Coverall	476	85.4
Safety shoe	340	61.3
Glove	200	36.0
Respirator	110	19.8
Helmet	30	5.4
Ear plug	24	4.3
Drink alcohol		
Yes	322	58.0
No	233	42.0
Smoke cigarette		
Yes	244	44.0
No	311	56.0
Chew khat		
Yes	219	39.5
No	336	60.5

### Work-related health complaints

Two-thirds, 66.8 %, of the workers reported they experienced at least one health complaint related to their work. The most common complaints were 99.6 % vision problems, 94.2 % injuries to the body, and 54.1 % back pain (Table 3).

**Table 3:** Health complaints reported by welders at Lideta Sub-City, Addis Ababa, Ethiopia, 2015

Variables	Number	Percent
Vision problems		
Yes	553	99.6
No	2	0.4
Breathlessness		
Yes	166	29.9
No	389	70.1
Chronic cough		
Yes	12	2.2
No	543	97.8
Metallic fume fever		
Yes	20	3.6
No	535	96.4
Injuries to the body		
Yes	523	94.2
No	32	5.8
Back pain		
Yes	300	54.1
No	255	45.9
Hearing impairment		
Yes	69	12.4
No	486	87.6

### Participants’ awareness of occupational hazards

The majority, 86.5 %, of participants were aware of occupational hazards that might occur during the welding process. The highest level of awareness was observed among participants of 94.9 % large scale industries followed by 86.2 % medium scale and 83.5 % small scale.

### Factors associated with awareness of occupational hazards

Work experience, employment pattern, marital status, educational status, khat chewing, cigarette smoking, job satisfaction, safety training, supervision, work regulation, and health complaint showed significant association with awareness in the bivariate analysis. However, only work experience, job satisfaction, work regulation, marital status, and educational status remained significant in the multivariable logistic regression model (Table 4).

**Table 4:** Factors associated with awareness of occupational hazards among welders in Lideta Sub-City, Addis Ababa, Ethiopia, 2015

Variables	Hazard awareness		Crude OR (95 % CI)	Adjusted OR (95 % CI)
	Yes	No		
Work experience (in years)				
<5	118	33	1.0	1.00
5–9	179	29	1.7(0.1, 3.0)	2.7(1.3, 5.6)
10–14	91	8	3.2(1.4, 7.2)	5.6(1.7, 18.8)
≥15	92	5	5.1(1.9, 13.7)	2.4(0.6, 9.9)
Work regulation				
Yes	305	25	3.5(2.1, 5.8)	2.4(1.1, 5.2)
No	175	50	1.0	1.0
Job satisfaction				
Yes	381	19	11.3(6.4, 20.0)	9.3(4.3–20.1)
No	99	56	1.0	1.0
Marital status				
Married	239	32	18.7(6.8, 51.6)	12.6(3.4, 46.6)
Single	235	28	21.0(7.5, 58.4)	11.4(3.1–41.9)
Divorced	6	15	1.0	1.0
Educational status				
Primary	62	21	1.0	1.0
Secondary	207	37	1.9(1.0, 3.5)	2.8(1.2, 6.6)
Certificate and above	211	17	4.2(2.1, 8.5)	2.7(1.1, 6.7)

## Discussion

Welding is a manufacturing industry where workers could be exposed to several hazards, like fumes and gases, dust, intense bright light, excessive noise, vibrations, electricity, intense heat, unsecured gas cylinders, awkward work postures, and fast moving machinery such as grinders. Proper awareness of these hazards is important to design safety education programs, use the different protective devices, and train in ergonomics and appropriate design of tools and machines to achieve greater efficiency of both man and machine. In this study 86.5 % of the workers were observed to be aware of the existence of different hazards related to their work. This finding is slightly different from studies reported from Nigeria (77.9–91.6 %) [5, 17]. The discrepancies between studies could be due to methodological differences, like study population, definitions of hazard awareness, methods of data collection, and workplace conditions.

This study identified important predictors influencing workers awareness of hazards related to their work. The odds of hazard awareness among employees who had longer work experience were nearly six times higher compared to those who served for less than five years. The possible explanation for this may be that those workers who served longer could have good knowledge and skills on machines and tools in use and become familiar to the work environment. In addition to this they might be exposed to different safety training sessions that could improve their awareness.

In this and other studies workers hazard awareness was found to be significantly associated with the presence of workplace safety regulations [13]. It is a fact that proper implementation of workplace safety regulations could help to monitor workers behavior and allow them complies with the safe procedures of their jobs. This, in proper integration with other safety programs, is a good strategy to mitigate safety culture impediments and to enhance workers awareness of occupational hazards.

Another important finding of this study was that the odds of hazard awareness among employees who were satisfied in their jobs were more than nine times higher compared to those who were not. An increasing number of studies have considered job satisfaction as pervasive and influential factor in the prevention of workplace hazards [18]. This could be linked to fact that when workers were satisfied in their jobs, they could experience meaningfulness, greater responsibility, and better use of their knowledge and skills in their jobs. Increased job satisfaction could lead to greater attention to safety motivation, knowledge, and compliance [19].

Higher odds of hazard awareness were observed among married [20] and single workers compared to those who were divorced in this study. This could be to the fact that those who were divorced could be worried with unrelated issues and might not give an attention towards their safety. This striking difference warrants further investigation on whether this group may be less attentive towards personal safety.

Workers’ awareness of occupational hazards was dependent on their increased level of educational attainment. This is in agreement with study conducted in Nigeria [5]. This might be due to the fact that workers who attained a higher level of education could have the tendency to change available information into mature stage which increased their awareness of hazards.

Social desirability bias is a potential limitation in self-reported studies like this one, in that employees might report more socially acceptable responses than their actual day to day practice. As this is a cross-sectional study, the limitations that come with this type of design need to be taken into consideration when interpreting the findings.

## Conclusion

This study revealed that the level of awareness of occupational hazards among welders was high.

However, this does not mean that there will be no need for further strengthening of the safety measures as significant proportions of the workers still had low awareness. Interventions to boost workers awareness of occupational hazards should focus on areas, such as provision of safety trainings, promotion of safety advocacy, and enforcement of appropriate workplace safety regulation.
